# X-ray data reconstruction from incomplete data sampling

**DOI:** 10.1107/S1600576725010003

**Published:** 2025-11-26

**Authors:** Kárel García Medina, Ernesto Estevez Rams, Reinhard B. Neder

**Affiliations:** a Lehrstuhl für Kristallographie und Strukturphysik, Friedrich-Alexander-Universität Erlangen-Nürnberg, Germany; b Faculty of Physics, University of Havana, Cuba; Oak Ridge National Laboratory, USA

**Keywords:** X-ray diffraction, sampling, Papoulis–Gerchberg

## Abstract

For specific setups, a diffraction pattern can contain gaps or missing information. For example, that is the case when several detectors are used simultaneously, but a particular angular range is not covered between each detector. In this article, a procedure for the reconstruction of the missing signal is proposed. It is based on a modified Papoulis–Gerchberg algorithm that considers features of the diffraction pattern without loss of generality. The mathematical basis of the algorithm is presented, and several cases, simulated and experimental, are used to test the performance and robustness of the proposed solution.

## Abstract 2

A reconstruction procedure for patterns with incomplete information is reported. It is based on a modified Papoulis–Gerchberg algorithm. The modifications were introduced to guide the reconstruction procedure. The reconstruction algorithm could prove useful in specific experimental geometry setups.

## 1. Introduction

There are several cases where the sampling of diffraction patterns is far from ideal, resulting in uneven data collection or gaps in the collected data. Such is the case, for example, when several line detectors are used simultaneously to improve the time resolution of the experiment but between each detector there is an angular range that is not covered. When faced with such settings, an alternative could be to repeat the experiment in different geometric configurations to fill in the missing information or reconstruct the whole signal from a single measurement. The former loses the advantage of speedy collection of the data, and for the latter its robustness and reliability must be proved.

Diffraction data with partially missing sections, like a powder diffraction pattern with gaps, can be treated without the need for data reconstruction if the data analysis involves the generation of a calculated diffraction pattern. This is, for example, the case for a Rietveld refinement. Individual sections of the powder diffraction pattern can even be adjusted with individual scale factors. However, the gaps need to be reconstructed if secondary data are to be calculated from the entire data set. A prominent case arises in the calculation of the pair distribution function (PDF) for powder diffraction. The required sine Fourier transform is severely affected by gaps in the original powder pattern, as we will present in a real example. While not treated in this manuscript, similar issues arise in the calculation of the three-dimensional difference pair distribution function from single-crystal data. In this contribution, we aim to reconstruct the whole signal from incomplete data and show how this can be done in a simple algorithmic manner with satisfactory results under certain constraints.

Signal reconstruction is a subgenre in the signal analysis literature starting from the seminal work that led to the Whittaker–Kotelnikov–Shannon sampling theorem or WKS theorem [for a historical perspective, see, for example, the book by Butzer *et al.* (2001 ▸) and references therein]. The theorem states that, if a band-limited signal is sampled fast enough, the whole signal can be reconstructed from the incomplete discrete data. Whether the sampling is ‘fast enough’ is related to the Nyquist frequency, which is double the largest frequency Ω in the Fourier spectrum of the signal. The theorem assumes that the signal is continuous and square-integrable, which is the case for most signals of interest.

The WKS theorem is constructive in the sense that it leads directly to the Shannon sampling formula for the reconstruction of the signal 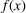, provided it has been sampled at equidistant points 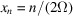: 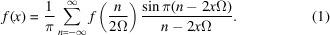
The practical use of the interpolation equation (1) has several limitations: We need infinite equidistant sampled points. Evenly distributed points can be a problem in some cases, but more seriously, the experimental data are always a finite subset of the needed infinite set of sampled data. The truncation of the series in (1) can lead to distortions of the recovered signal depending on the number of available data. Additionally, the Shannon interpolation formula can be very inefficient from a computational perspective. These limitations have led to the search for other interpolation procedures that better reconstruct the signal given the finite number of sampled points (Zayed & Butzer, 2001 ▸).

Regarding the limitation of the unevenness of the sampled points, various approaches have been developed (Feichtinger *et al.*, 1995 ▸; Strohmer, 2000 ▸; Marvasti, 2001 ▸; Lacaze, 2001 ▸; Hyberts *et al.*, 2012 ▸; Lu & Xian, 2020 ▸). Recently, several procedures referred to as compressed sensing have gained wide attention due to their robust nature (Donoho, 2006 ▸; Candes *et al.*, 2006 ▸). The surprising aspect of compressed sensing is that, under certain conditions of sparsity in a chosen space, it can recover a signal even if the data are sampled below the Nyquist rate. These methods really shine with random information loss while only assuming sparsity priors (Hyberts *et al.*, 2012 ▸; Butzer *et al.*, 2001 ▸; Lacaze, 2001 ▸). They can be thought of as constrained optimization problems, with the associated computational cost. Despite the development of powerful optimization techniques like total variation minimization (Zeng, 2024 ▸; Han *et al.*, 2012 ▸), solving these complex non-linear optimization problems often requires expensive algorithms like the fast iterative soft threshold algorithm, the alternating direction method of multipliers or iteratively re-weighted 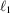 minimization (Wei *et al.*, 2022 ▸; Long *et al.*, 2021 ▸; Wang *et al.*, 2025 ▸).

A related but not equivalent problem is when the sampled data have continuous missing information, that is, a gap in the collection of the data. In such a case, the challenge of reconstructing the signal is even more significant. Compressed sensing methods are optimized for random, incoherent sampling which ensures that the missing data are not highly correlated or structured. This is not the case with gaps where there is a systematic loss of information over a continuous inter­val. When that is the case, other methods might be better suited.

Recent developments in data-driven methods, like deep learning reconstruction (DLR), have shown promise in addressing these challenges. These techniques learn implicit priors from large data sets through the use of deep neural networks (DNNs) (Rahman *et al.*, 2023 ▸; Koetzier *et al.*, 2023 ▸; Quaia *et al.*, 2024 ▸; Greffier *et al.*, 2022 ▸). DLR methods have proven effective and robust in reconstructions tasks, achieving high fidelity and low levels of noise (Koetzier *et al.*, 2023 ▸). Furthermore, once trained, DNNs efficiently map incomplete data to reconstructed signals in a non-iterative manner, making them suitable for real-time applications (Quaia *et al.*, 2024 ▸). There is, however, the drawback that they need large data sets for training, which might not be always available. When the problem at hand is related to highly specialized experimental setups, or highly specific phenomena, data availability becomes a serious limitation.

In this contribution, we consider the problem of a diffraction pattern that could or could not be evenly sampled but has gaps in the underlying continuous signal which render the available data incomplete. Compressed sensing methods, and the corresponding sparse reconstruction techniques, become a naturally attractive choice given the nature of the data. The presence of sharp, well-defined Bragg peaks in the diffraction pattern suggests that the signal is sparse in the frequency domain. However, we are dealing with a systematic absence of data over continuous intervals, which challenges the assumption of random incoherent sampling required by compressed sensing methods.

With all of the above in mind, we focus on a well-known reconstruction method called the Papoulis–Gerchberg procedure (Gerchberg, 1974 ▸; Papoulis, 1975 ▸) that has been used in several contexts, including the recovery of missing data (Ferreira, 2001 ▸; Hsu & Lo, 2005 ▸; Chatterjee *et al.*, 2009 ▸; Prieto-Guerrero *et al.*, 2009 ▸). This algorithm iteratively enforces two constraints on the data: known data values in direct space and band-limiting in the Fourier space. Since the algorithm operates on simple Fourier transforms and frequency filtering, it has a low computational cost per iteration when compared with other methods (Ferreira, 2001 ▸) and does not require huge amounts of data. However, its iterative nature means a finite, potentially large, number of iterations might be needed to reach convergence. More importantly, its convergence highly relies on the data adhering to the band-limiting assumption, which might not always hold true in practical scenarios. The fact that we are dealing with finite data in direct space means that the band-limiting assumption is not strictly met, which might cause the algorithm to become unstable or converge to non-physical solutions. For this reason we are proposing a modified formulation of the Papoulis–Gerchberg algorithm to take into account the particularities of diffraction patterns and effectively guide the reconstruction to physically meaningful solutions.

## 2. Papoulis–Gerchberg algorithm

The idea behind the Papoulis–Gerchberg algorithm is rather simple.

Consider a signal 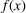, to be ideally sampled at discrete values 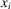, resulting in the vector 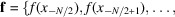

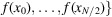
. The actual sampling, however, might be such that the sampled data have one or several gaps where the values of the functions are unknown.

The gaps can be mathematically represented by a mask vector, 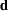, of length 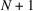, with zeroes in the positions of the gaps and 1s otherwise. A distribution of masks of width 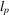 and centered on 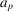 is the complement of the rectangular window, 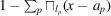 [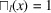 if 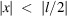, 0 otherwise].

The experimentally known data are then 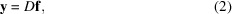
where *D* is a diagonal matrix with diagonal equal to 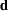.

Consider the discrete Fourier transform 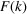 of 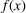, 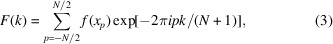
which can be represented in matrix form as 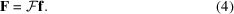
Here, 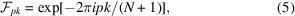
is the (symmetric, positive defined) 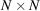 Fourier matrix. (The corresponding indexes *p* and *k* must be shifted by *N*/2.) It will be considered that the Fourier spectrum has finite bandwidth, that is, there exists a set *G* in *k* for which the Fourier components are zero [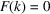, 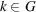], or, in our case, there exists a 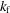 for which 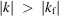 implies 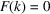.

The discrete inverse transform of 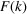 will be given by
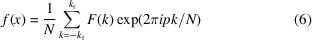
or
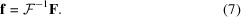
We can now write 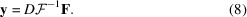
Let us introduce a value 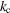 and define a vector mask γ in Fourier space such that it has values 1 in position 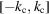 and 0 otherwise. The corresponding mask matrix Γ can be built following the same procedure for *D*. We now apply the Γ mask before the inverse Fourier transform:
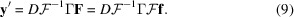
By definition, as long as 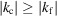, 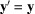, but this is not the case if 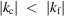. We call the matrix 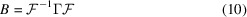
the band-limiting matrix or the Papoulis–Gerchberg matrix. The reader can check that 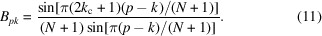
If the identity matrix is *I*, 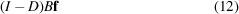
will give values in the gap and zero otherwise. Correspondingly, we define 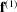, 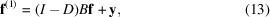
which will have the same values as *y* outside the gap interval and 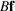 values in the gap interval. Papoulis–Gerchberg turned this into an iterative procedure:
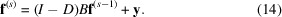
The procedure converges after a finite number of steps.

The interesting case is when 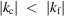. The band-limiting matrix is a low-frequency filter that smooths the signal by cutting high-order frequencies for such values. Each iteration step (14) then recovers the original values outside the gap and constructs a low-frequency (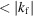) signal for the previous step in the gap interval. The iterative process is proven, for a well-chosen value of 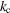, to be able to reconstruct the missing signal under certain conditions (Ferreira, 2001 ▸).

## 3. Modified Papoulis–Gerchberg algorithm

The effectiveness of the Papoulis–Gerchberg algorithm relies on the fact that the signal reconstructed for the gap has a Fourier spectrum that is band-limited close to the cut-off frequency 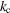. On one hand, if 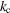 is well below the band interval of the signal in the gap then the reconstruction will miss harmonics needed in the reconstruction; if, on the other hand, it is well above the band interval, there will be several solutions to the reconstruction problem. Also, there is the implicit assumption that the missing information is still present in the Fourier spectrum of the experimental signal despite the gap.

**Figure 1:**
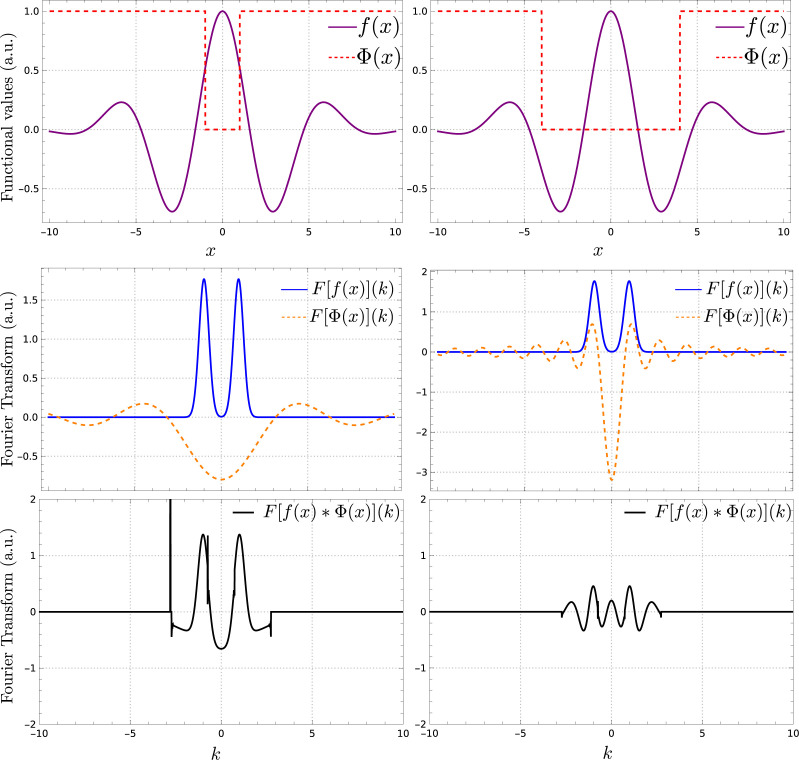
Schematic representation of gap effects. The top row shows a continuous function *f*(*x*) being filtered by a sampling function 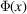, introducing a gap of two different sizes. All points outside the gap are sensed [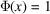], while those inside the gap are not [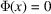]. The middle row shows the Fourier transform of the continuous function 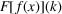 and the Fourier transform of the sampling function 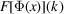, separately. The larger the gap in the *x* domain (top row, dashed line), the steeper the resulting Fourier transform becomes in the *k* domain (middle row, dashed line). This causes the convolution results in the bottom row to deviate more strongly from the Fourier transform of the original function *f*(*x*) (middle row, solid line).

To understand what the gap represents, consider a continuous function 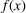 being sampled through a gapped sampling function 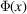, which is 1 outside the gap and 0 inside, as shown in Fig. 1 ▸ (top row). If the gap is small enough, its Fourier transform 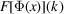 (Fig. 1 ▸, middle row) will be smooth enough to avoid mixing the contribution from neighboring Fourier components when convolved with the Fourier transform of the original signal 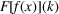 (Fig. 1 ▸, bottom row). On the other hand, if the gap is too large then the convolution will lead to some aliasing, mixing the contribution of neighboring Fourier components. The larger the gap, the greater the mixing in the Fourier landscape of the original function.

In light of the above considerations and the inherent characteristics of the processed signals, we implement a modified soft frequency filter. During each iteration of the Papoulis–Gerchberg algorithm, all Fourier components within the open interval 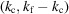 are set to zero. This approach permits controlled leakage of high-frequency components at every step, thereby enhancing the robustness of the reconstruction process. These high-frequency harmonics may, in principle, correspond to characteristic features of powder diffraction, such as extremely narrow Bragg reflections or phase mixtures, without being exclusively attributed to the gap.

**Figure 2:**
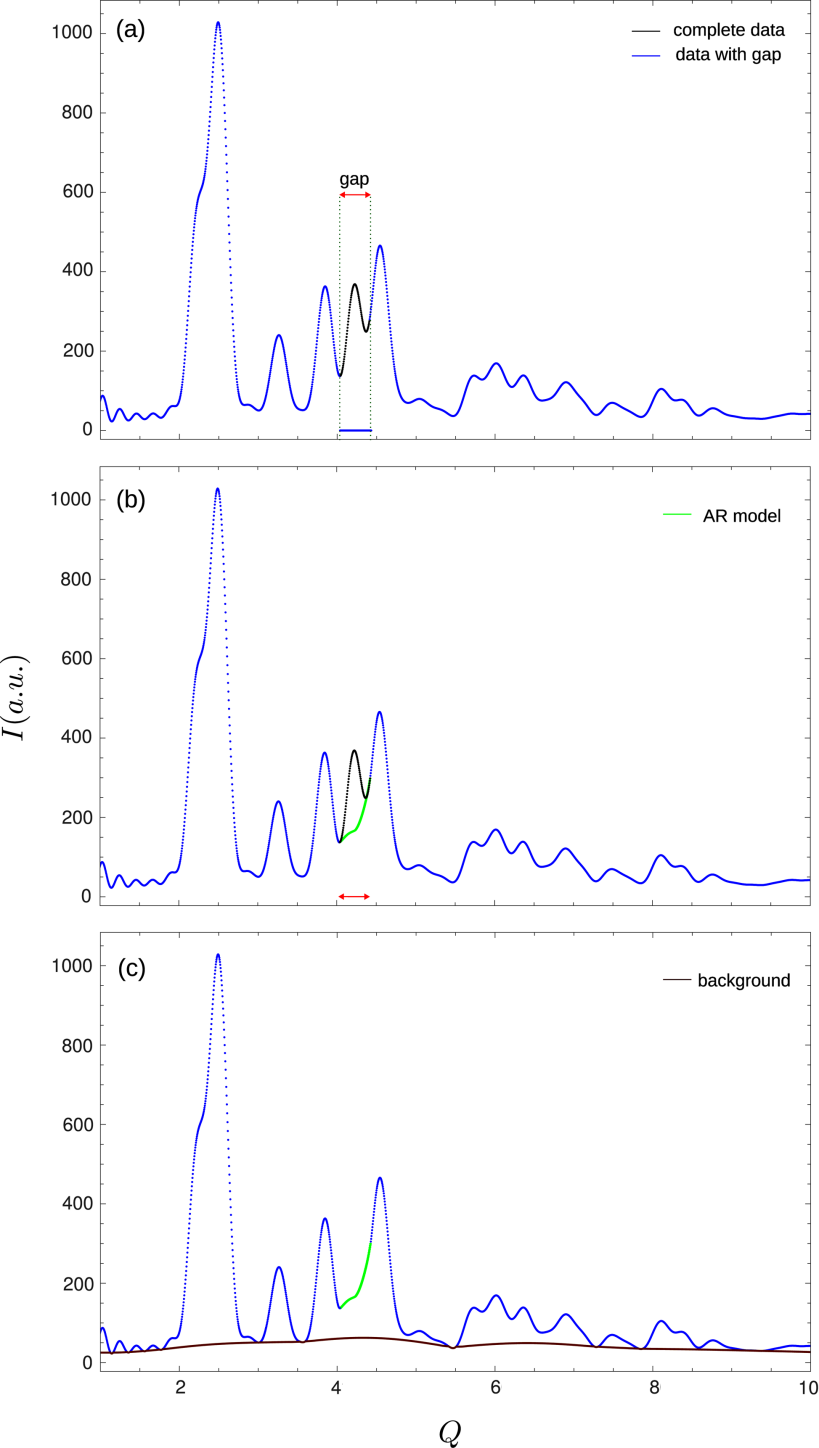
Preprocessing of the data. (*a*) The real (complete) data are not known (black). The available data (blue) have a gap that can hide relevant information, such as a peak. (*b*) The gap in the available data (blue) is estimated by an AR procedure (see text) that results in a continuous curve (green) bridging the two sides of the data. The AR estimation does not need to resemble the actual signal in the gap. (*c*) The lower envelope is estimated as a smooth curve from the now continuous signal. Any background procedure can be used. In the reported results, a low-frequency filter background estimator was applied (see text).

Furthermore, we incorporate two additional modifications to the method to better guide the reconstruction. Consider the experimental signal **y** given by equation (2). The first change is that, instead of starting with the signal [Fig. 2 ▸(*a*)] taking the gap values to zero, an autoregression model (AR) is used [Fig. 2 ▸(*b*)] from the left and from the right to build as a starting point a continuous signal within the gap. This has been done before as a signal recovery technique in the context of audio and ECG signals (Esquef *et al.*, 2003 ▸; Prieto-Guerrero *et al.*, 2007 ▸). In the present paper, we use it as an initial step before using the Papoulis–Gerchberg algorithm.

The autoregressive model of a signal 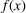 is defined as (Kay, 1999 ▸) 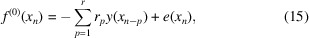
where *p* is the model order, which can be automatically determined by several means; here, the Akaike information criterion is adopted (Stoica & Selen, 2004 ▸). *r*_*p*_ and *e*(*x_n_*) represent, respectively, the coefficients and residuals obtained from fitting the model to known data (outside gapped regions). If the autoregression model is correctly fitted, and *r_p_* are optimal, the residuals defined by *e*(*x_n_*) will be white noise. The modeling is used to interpolate, via both backward and forward prediction, in the missing part of the signal. The forward and backward models are then added using a cross-fading window to guarantee the continuity of the whole reconstruction.

The used cross-fading window is given by
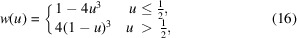
where 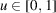.

The autoregression model is not sufficient for signal reconstruction as it cannot reconstruct missed Bragg peaks if the gap is wide enough to erase the whole peak in the sampled signal.

The second step is to determine, from the sampled signal 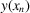, the lower envelope [Fig. 2 ▸(*c*)]. Such steps will be used during the iterative process to avoid unrealistic signal reconstruction, such as negative values or values well below the signal background. Here, several background estimations can be used, and in what follows, the procedure described by Brückner (2000 ▸) was adopted.

**Figure 3:**
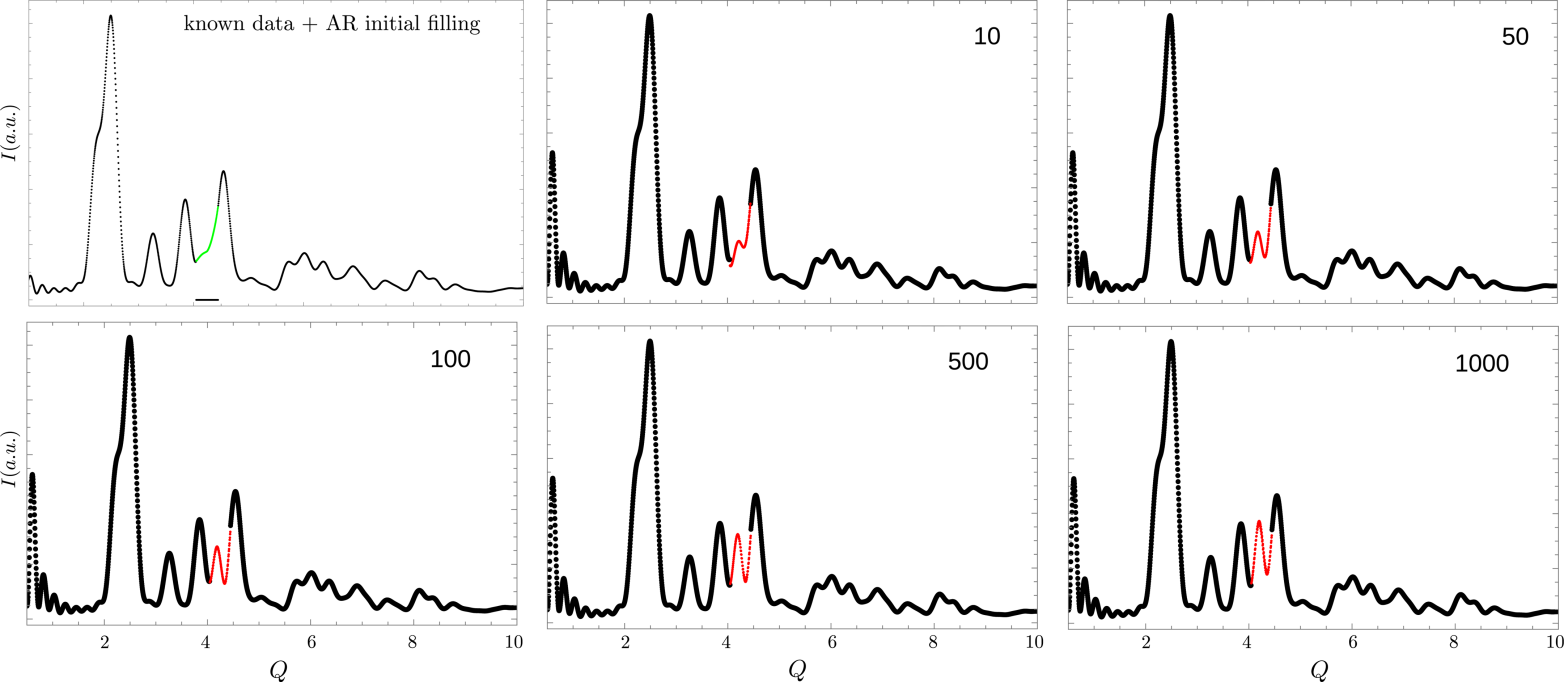
Iterative process. Starting from the AR-filled signal, the modified Papoulis–Gerchberg algorithm is applied. The algorithm is iterative in nature, and it can be seen that, as more iterations are performed, the reconstructed signal is approached. After cutting the spectrum in the signal’s Fourier transform, the known experimental points are reimposed in the inverse transform data, and the next step starts. The iteration can be stopped when two successive steps do not significantly change the reconstructed signal. The reconstructed signal can be seen in the figure after 10, 50, 100, 500 and 1000 iterations. No significant changes were perceived after 1000 steps.

With the lower envelope estimate 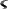, the modified Papoulis–Gerchberg iterative step (14) is changed to 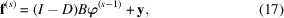
where 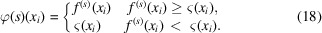
For a well chosen cut-off frequency, 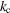, the iteration converges to the reconstructed signal (Fig. 3 ▸). The selection of the cut-off frequency will be discussed below.

Iterations are stopped when the difference between the current and previous steps is below a certain threshold:
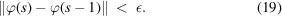
The final solution 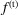 is then taken as the output.

**Figure 4:**
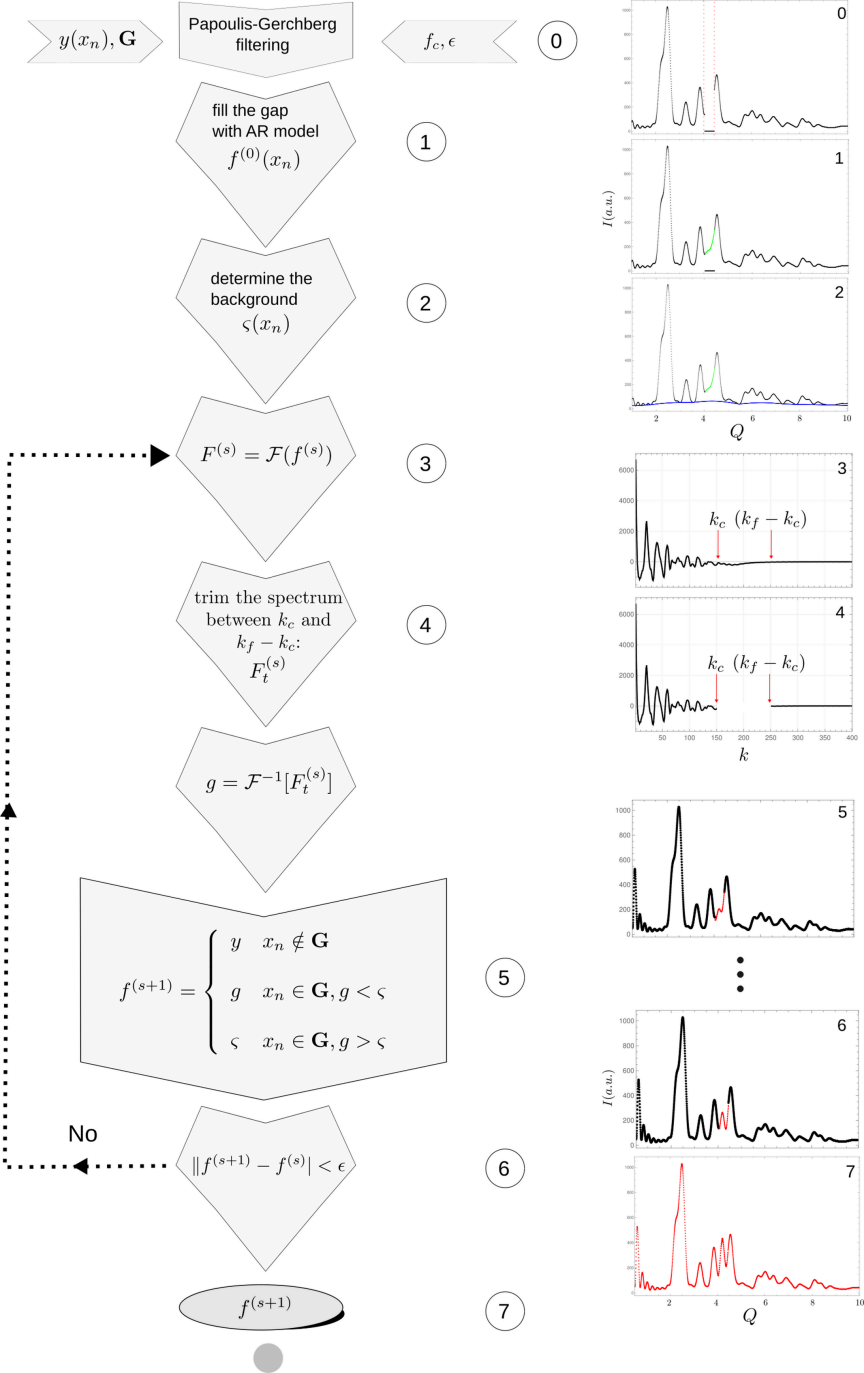
Flow diagram of the modified Papoulis–Gerchberg reconstruction algorithm. The input data consist of the incomplete signal, together with the information regarding the gap position as a mask (*G*), the cut-off frequency 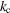 and the stop criterion as an ε value, which is used as a threshold to compare changes in two successive steps. The gap is filled with the AR model (1) and the lower envelope is estimated (2). The modified Papoulis–Gerchberg iterative procedure is then performed (3–5) while the difference between two successive reconstructed signals is above the threshold ε (6). The obtained signal is taken as the reconstructed result when the threshold is reached.

The idea behind these modifications is to improve the reconstruction process in a highly pathological solution space. Fig. 4 ▸ summarizes the steps of the iterative algorithm.

### 3.1. Choosing the cut-off frequency

Choosing the appropriate control parameters in optimization problems is far from a trivial task and one that is not completely solved. As mentioned by a number of authors (Eilers, 2017 ▸), without the target data there is no guarantee, in general, that a given procedure will result in obtaining as solution the global minimum. The compromise is to settle for getting a ‘good enough’ solution. This will be our goal in introducing several figures of merit to determine the appropriate cut-off frequency.

The cross-validation error (CVE) (Eilers, 2003 ▸) measures the difference between the reconstructed and original signals. The signal 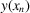 is randomly sampled to a percentage lower than 1, and a new signal 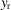 is obtained. Reconstruction is then performed to 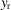 for a given cut-off frequency value, and the reconstructed signal 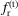 is compared with the original signal *y*. The CVE is then defined as 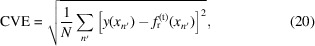
where 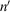 means the sum is carried out over all points outside the gap.

Only non-sampled points outside the gap will contribute to the CVE. Thus, the smaller the CVE, the better the reconstruction in terms of the known data representation. The process is repeated several times to get an average value of the CVE for the given cut-off frequency value.

The overall normalized error (ONE) was determined between the final inverse Fourier transform in the Papoulis–Gerchberg algorithm (*g* in Fig. 4 ▸) and the original signal (*y* in Fig. 4 ▸). As part of the algorithm, known values are imposed on the recovered signal when returning from Fourier space to direct space at each iteration, thus guaranteeing equality of those points. One could expect, however, that the better the choice of the cut-off frequency, the better the reconstruction. That is, the better the choice of the cut-off frequency, the more similar would the final recovered signal be to the original one, even before equality is imposed on the known points. The total absolute value of the difference was then calculated and normalized by the number of known points to get 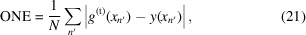
where *g*^(t)^ represents the final output of the iterative algorithm (upon convergence) before equality is imposed on known data points (see Fig. 4 ▸). ONE is thus another measure of how good the reconstruction is in terms of known signal representation.

Finally, borrowing from compressed sensing (Candes *et al.*, 2006 ▸), frequency sparsity (FS) is defined. The frequency value 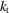 for which the power spectrum of the Fourier transform of the reconstructed signal (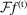) reaches a given threshold is used as a criterion. The criterion for better reconstruction is then taken as the cut-off frequency that results in the smaller 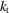 . This can be thought of as the simplest solution, in terms of frequency components, that still represents the known data.

In order to compare the reconstructed signal 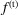 with the original one *f* (without gap) the mean squared error (MSE), not available in practical cases, was calculated as a natural measure of reconstruction quality:
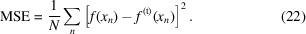


## 4. Results

**Figure 5:**
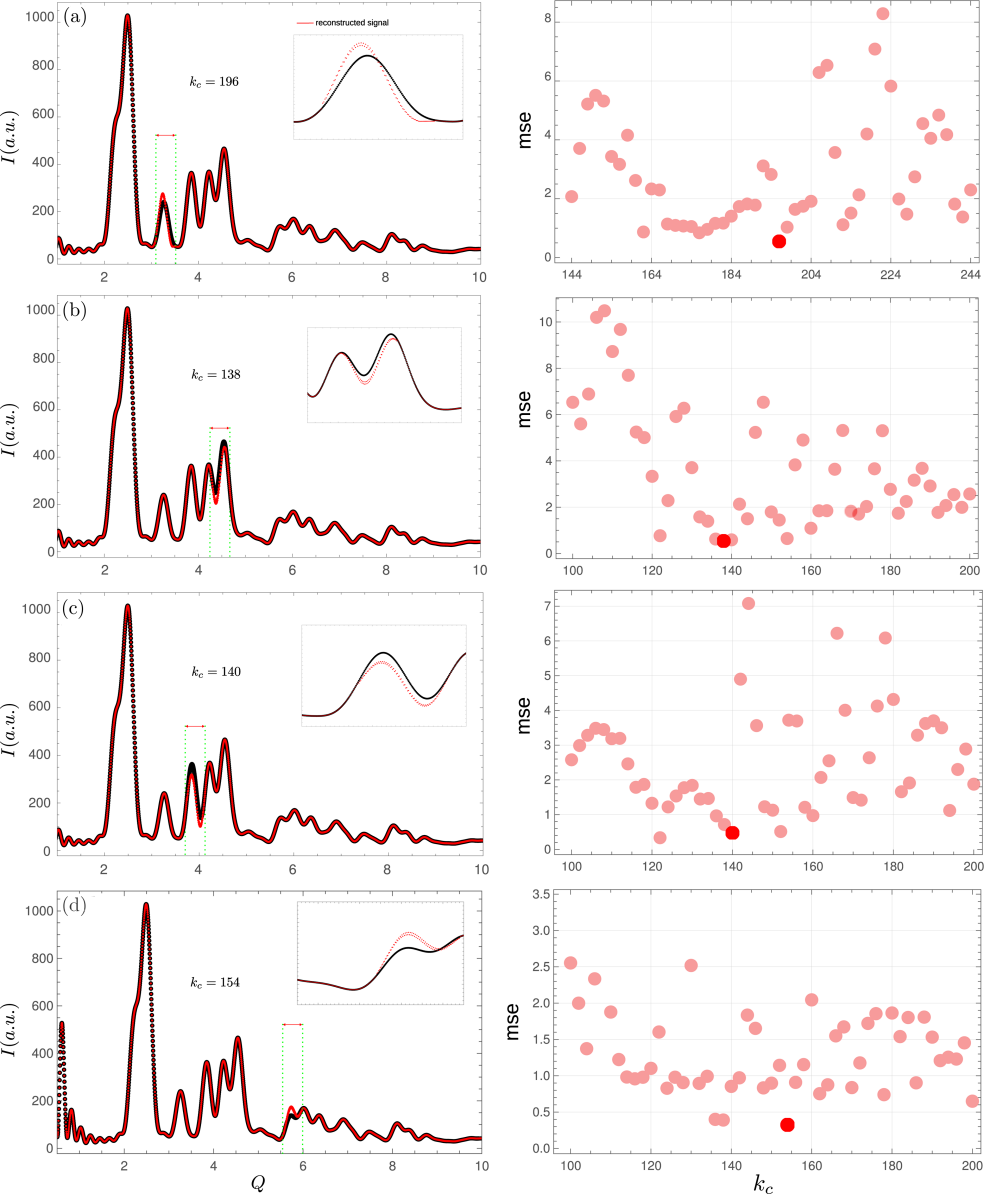
Reconstruction of simulated signal with the gap at different positions. In all cases, the gap size is 100 *Q* values and the reconstruction corresponding to the minimum square error is taken. Insets show gapped regions more closely. The reconstructed signals show that the algorithm proves robust to the gap position, though highly sensitive to the cut-off frequency.

A simulated diffraction pattern was generated for a ZnO nanocrystalline sample using the *DISCUS* suite of programs for diffraction (Neder & Proffen, 2008 ▸; Proffen & Neder, 1999 ▸). A 100-*Q*-value gap was taken at different positions of the diffraction pattern. Fig. 5 ▸ shows the reconstruction results. For Figs. 5 ▸(*a*)–5 ▸(*c*), the gap is missing entirely or partially a single Bragg reflection (*a*) or more than one (*b*–*c*). The better reconstruction regarding the lower value of MSE is chosen in all the examples. Despite the gap, the recovered signal completely reconstructs the missing peaks with a slight shift in position and intensity. Examples (*b*) and (*c*) are noteworthy as the peak position has a relative shift of less than 1%. For Fig. 5 ▸(*d*), the gap is chosen for larger values of *Q* where the signal intensity for the Bragg peaks is already much lower than that found for small *Q* values. The iterative algorithm again succeeds in reconstructing the peak, but the relative error is higher than in the previous cases. However, the reconstruction performed quite robustly in this less favorable case. Nevertheless, inspection of the right-hand mean squared error plots shows that the algorithm is highly sensitive to the cut-off frequency value, with several local minima present and highly structured behavior.

**Figure 6:**
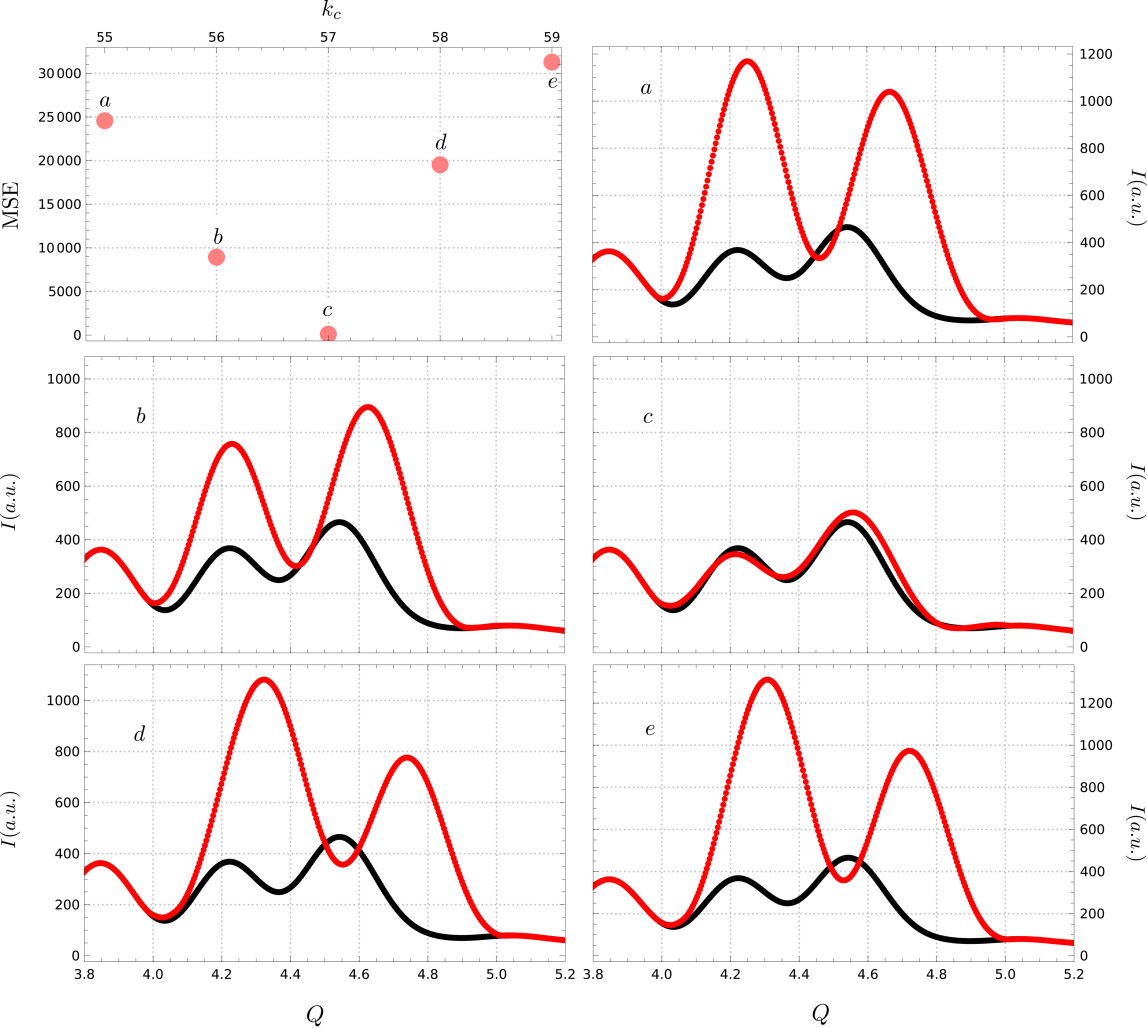
Reconstruction sensitivity to cut-off frequency. The locally optimal cut-off frequency was found to be 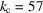 (*c*), with neighboring frequencies (*a*, *b*, *d*, *e*) leading to drastically worse reconstructions, both visually and quantitatively.

For context, Fig. 6 ▸ shows the reconstruction results for the same simulated pattern with the gap in roughly the same position as in Fig. 5 ▸(*b*) but much broader now, covering the 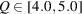
 Å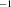 range. The locally optimal cut-off frequency was found to be 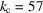, with the MSE plot showing a local minimum and the corresponding reconstruction labeled *c*. However, note the drastic jump in MSE values for both neighboring cut-off frequencies, 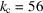 and 58, and further. The jumps are of the order of 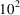 [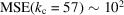]. The algorithm’s sensitivity is further confirmed by the corresponding reconstructions labeled *a*, *b*, *d* and *e*. This further highlights the need for guided selection of the cut-off frequency value when dealing with highly pathological solution spaces, like the one at hand.

**Figure 7:**
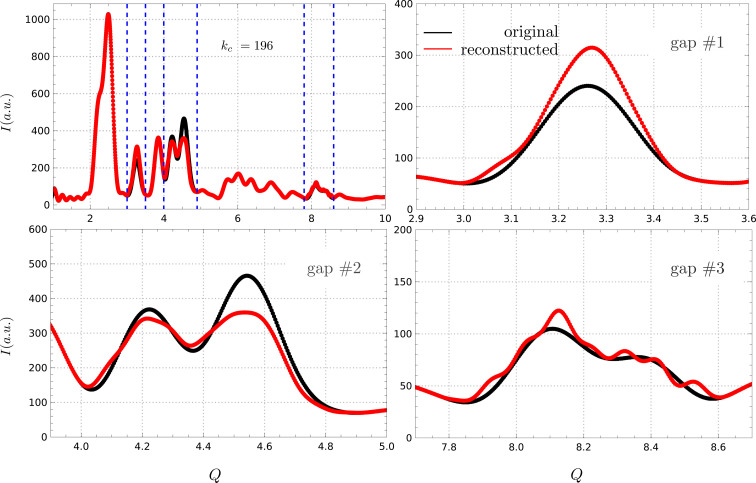
Reconstruction with multiple gaps. The algorithm proved surprisingly robust to the presence of multiple gaps in the data, with some intensity discrepancies (gaps #1 and #2) and aliasing effects (gap #3).

**Figure 8:**
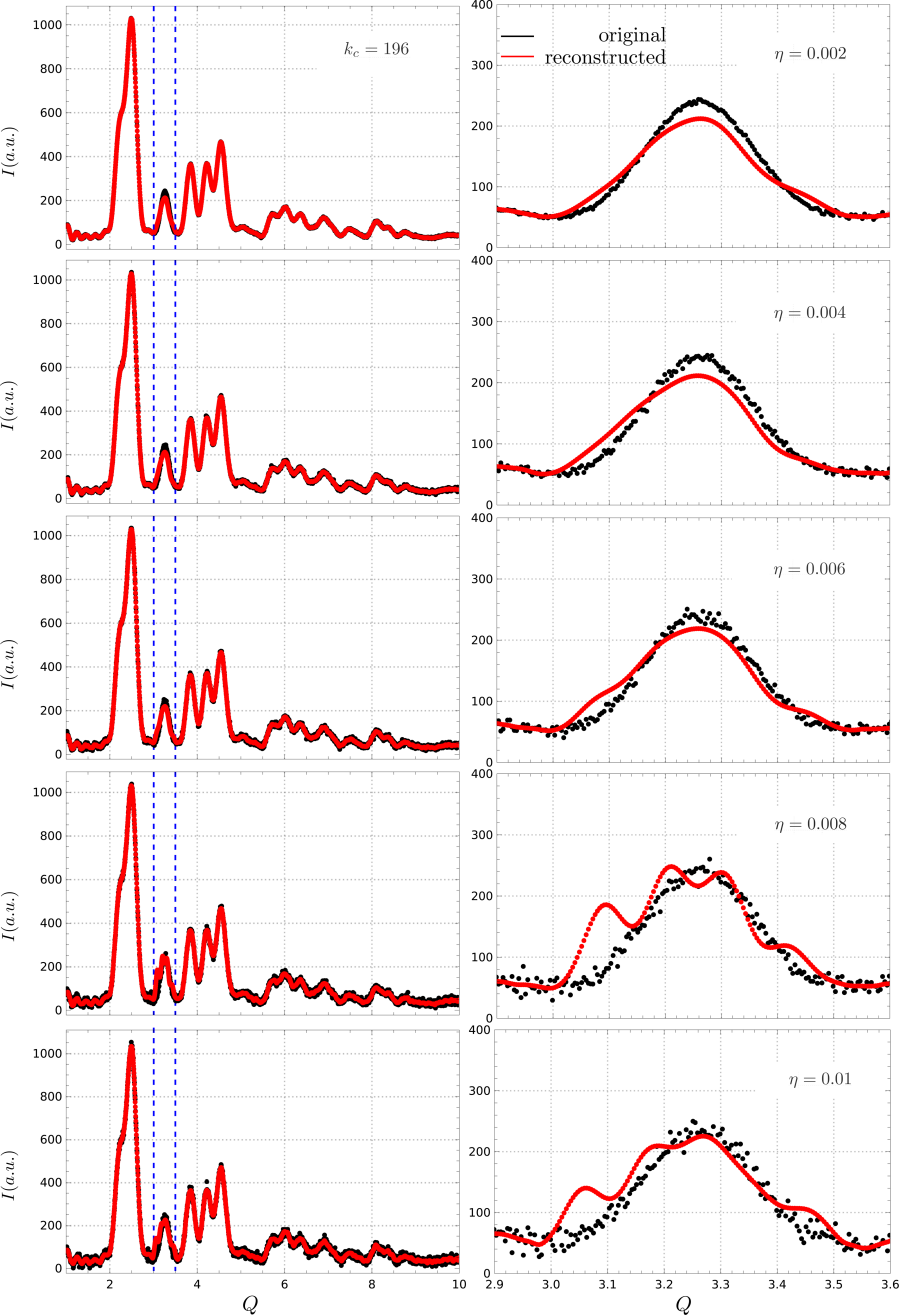
Reconstruction with different noise levels. The algorithm demonstrated robustness across reasonable noise levels, although some aliasing effects were observed at higher noise levels.

Next, the same simulated pattern was used to test the reconstruction procedure in the presence of multiple gaps (Fig. 7 ▸) and different noise levels (Fig. 8 ▸) to explore the algorithm’s robustness. The gaps where chosen at different positions and with different sizes, sometimes removing multiple Bragg reflections. The reconstruction was made with 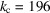, which showed decent performance when reconstructing gap #1 in Fig. 5 ▸(*a*). The algorithm proved surprisingly robust, effectively reconstructing all missing peaks, despite some aliasing effects being visible in gap #3 and some intensity discrepancies.

The noise, on the other hand, was modeled as a zero-mean Gaussian noise with a standard deviation proportional to the maximum intensity of the signal times a factor η, thus simulating different signal-to-noise ratios. The reconstruction was performed with 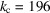 in all cases, while the algorithm proved robust for all reasonable noise levels. For 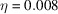, aliasing effects become visible. Good statistics are thus advised when applying the reconstruction procedure, especially since it is based on soft frequency filtering, which can be highly sensitive to high-frequency noise.

**Figure 9:**
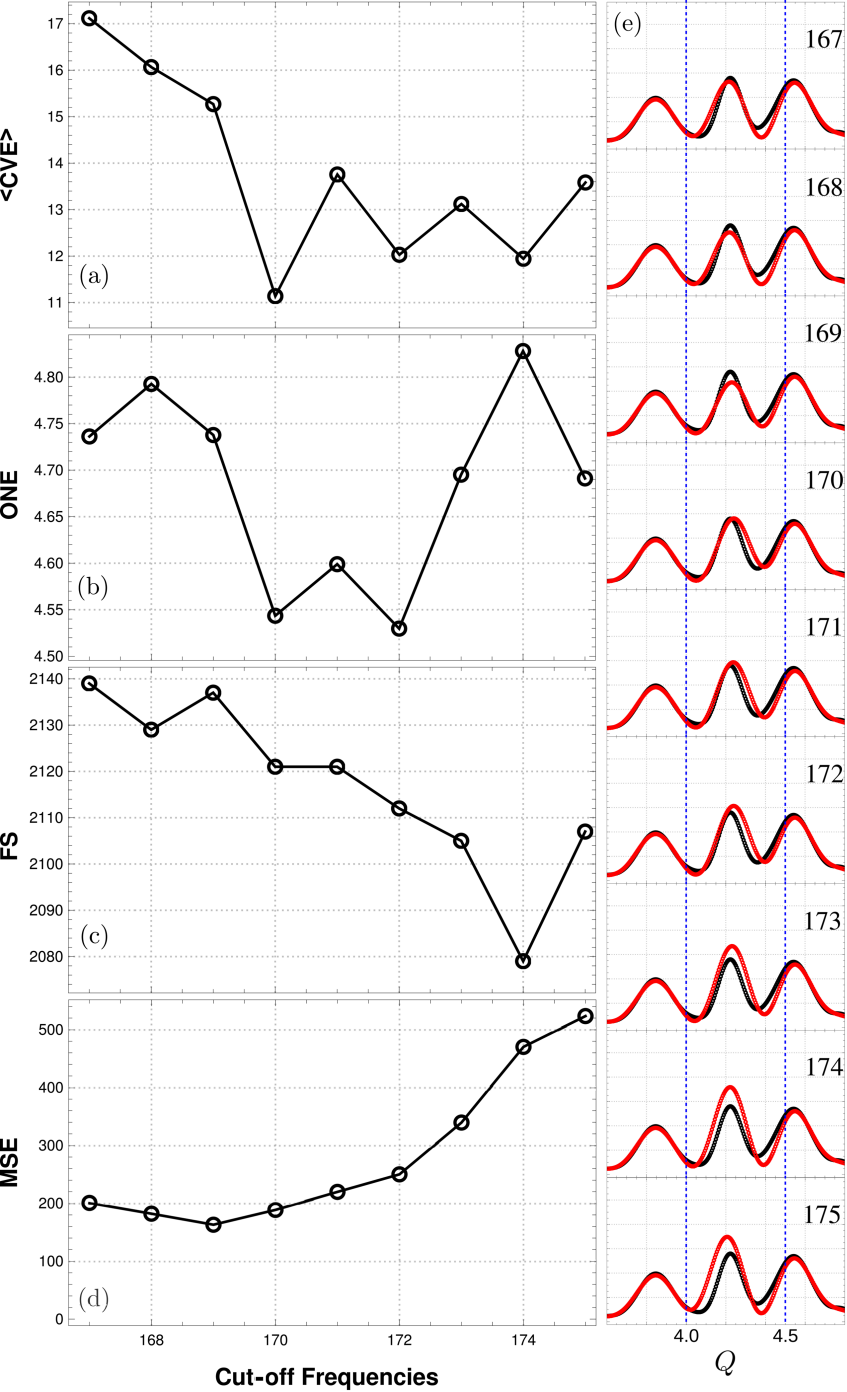
Metrics to choose the cut-off frequency. Three practical metrics have been defined that can be derived from the observed and reconstructed data. Cross-validation error was calculated for several instances of random sampling, and the average, 〈CVE〉, is reported (*a*). (*b*) As explained in the text, the overall normalized error (ONE) is determined between the final output of the iterative algorithm (*g*^(t)^) and the original signal (*y*). The third metric, FS (*c*), is taken as the frequency value for which the power spectrum of the Fourier transform of the reconstructed signal reaches a given threshold. According to the MSE (*d*), the best reconstruction was attained with 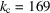. Reconstructed signals for values around the MSE minimum are shown in the right column (*e*). 〈CVE〉 has a minimum at the neighboring point 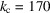, leading to the second-best reconstructed signal near the absolute MSE minimum. The same 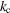 value is one of the two minima in the ONE figure of merit, while FS has a minimum value for 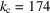, where the reconstruction is still visually close to the original signal despite not being near the cut-off frequency at which MSE reaches its minimum value.

Fig. 9 ▸ shows the different metrics and the reconstruction output for the same simulated data for a range of cut-off frequencies. According to the MSE value, the best cut-off frequency is 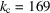, with 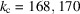 close to the minimum. 〈CVE〉 and ONE have a minimum for 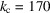. The later also has a minimum at 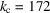 which corresponds to the fourth-best value of MSE. The sparsity criterion does not perform well, having a minimum at 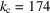 which is the eighth-best value of MSE. This type of analysis was performed for several gap positions; in all cases, the best MSE value was among the smallest four values of 〈CVE〉 and ONE. Also, the minimum value of the used metrics leads to a good reconstruction according to the MSE metric in all cases. This seems to point to the idea that, in practical cases where the original signal is not known, a combination of 〈CVE〉 and ONE can be used to guide the selection of locally optimal cut-off frequency values. As already seen in Fig. 5 ▸, the high sensitivity of the algorithm to the cut-off frequency causes the metrics plotted in Fig. 9 ▸ to be highly structured, with potentially several local minima.

**Figure 10:**
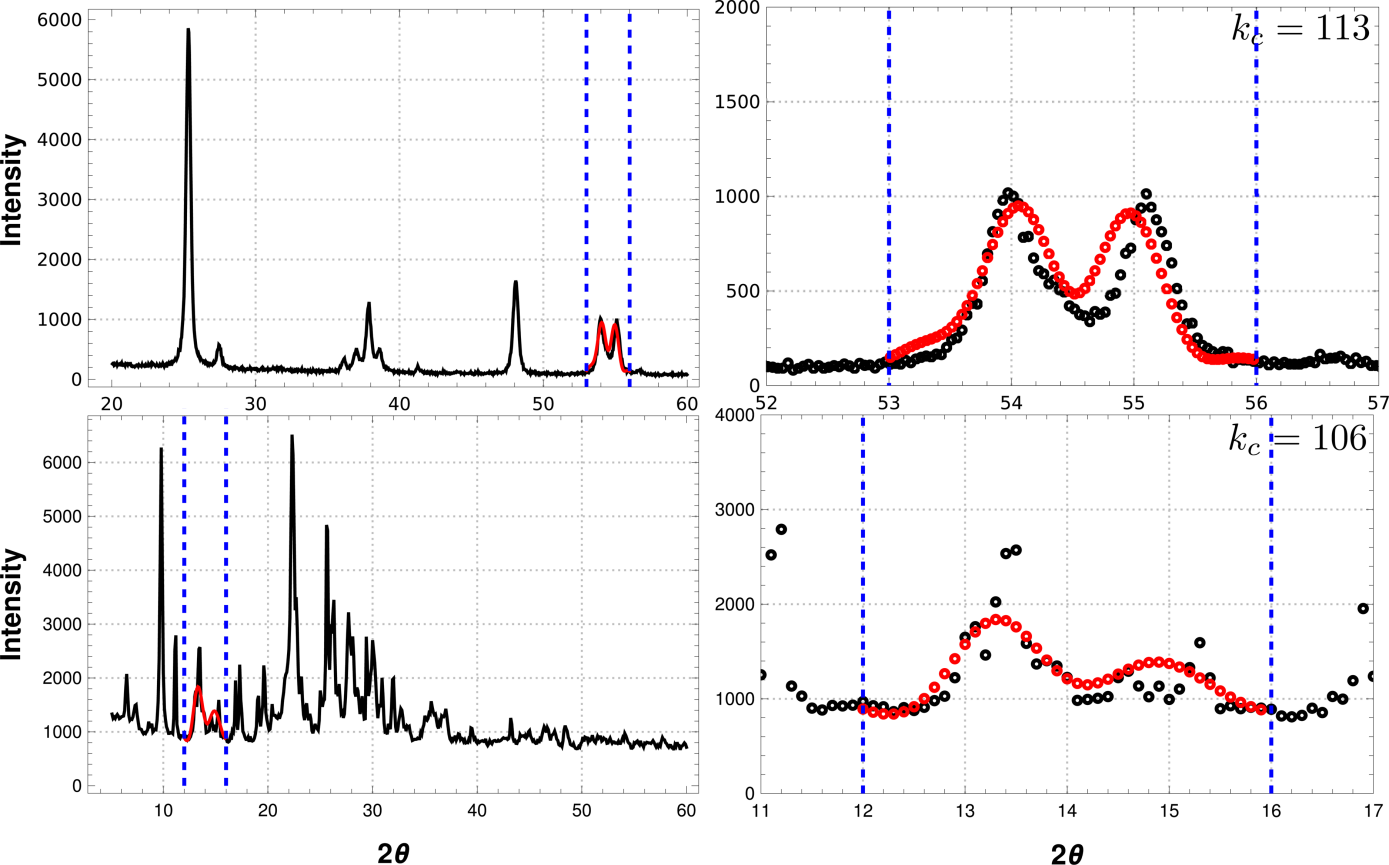
Experimental results. Two experimental data sets were used to evaluate the performance of the reconstruction procedure. The artificial gap is shown as vertical dashed lines. The upper panels are from a TiO_2_ (anatase) sample. The iterative algorithm can reconstruct the two peaks left out by the gap. The reconstructed signal is shown in red. The right-hand plot shows a zoom of the reconstructed region, where it is clear that the missing peaks have been recovered. The lower panels are from a pathological noisy sample with a mixture of phases clinoptilolite + mordenite + quartz. The gap was taken to shadow three smaller peaks in the data. In spite of the bad quality of the data collection, the algorithm is able to reconstruct the larger peak and another peak convoluting the other two smaller reflections. There is no hint whatsoever about the missing peaks in the data outside the gap, yet the algorithm is able to recover them in both cases.

Finally, the reconstruction procedure was applied to two experimental data sets, where an artificial gap was made. Pattern A (Fig. 10 ▸, top) is from a nanocrystalline TiO_2_ sample (experiment carried out on an XPERT PRO diffractometer with Cu *K*α source). The gap was placed in the interval [53.0, 56.0] of the 2θ axis, where two peaks are present. For the CVE, the minimum value is reached at 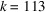, with the algorithm being able to reconstruct both peaks. Several 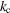 values around the chosen one effectively defined a set of acceptable solutions. Pattern B (Fig. 10 ▸, bottom) is from a sample where a mixture of at least three phases (clinoptilolite + mordenite + quartz) is present (experiment carried out on a Bruker D8 Focus with Cu *K*α source). This case can be pathological for two reasons: non-negligible noise on the pattern and the mixture of phases, which means that the Fourier transform of the pattern signal does not correspond to a single-crystalline phase. Yet, the algorithm can reconstruct the two main peaks in the gap region. More importantly, note how there are no hints of the missing features in the sensed data outside the gaps. In general, as shown for the synthetic data, reconstructions can be made for several gap lengths and positions, ultimately affecting the optimal cut-off frequency value.

When faced with incomplete experimental powder data, Rietveld refinements can still be done against partial patterns, thus avoiding the gapped regions. This would still allow for extraction of relevant structural information from the positions and intensities of the Bragg peaks outside the gap, related to long-range order in the sample (Dinnebier & Billinge, 2015 ▸). However, when dealing with amorphous or nanocrystalline materials, it is the diffuse scattering that contains the information on short-range order and the PDF analysis that can provide relevant structural information (Neder & Proffen, 2008 ▸). When that is the case, the entire pattern is needed. In a recent paper by Sapnik *et al.* (2025 ▸), high-quality ultra-fast total scattering and PDF data were obtained at an X-ray free-electron laser (XFEL) source. The study represents a major breakthrough as the authors demonstrated that their optimized experimental setup can collect high-quality total scattering data, for a wide range of materials, from a single roughly 30 fs X-ray pulse. The setup used a combination of overlapping Varex and JUNGFRAU area detectors to optimize the *Q* range coverage, which allowed for an uninterrupted 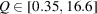
 Å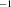.

**Figure 11:**
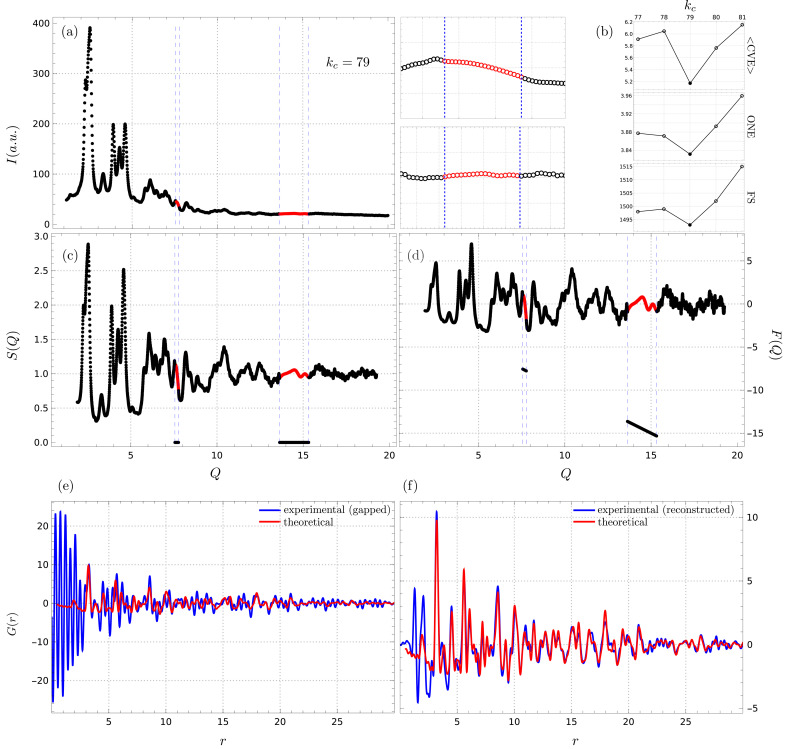
Experimental PDF results. (*a*) A gapped, and correspondingly reconstructed, pattern collected at the European XFEL source in a multi-JUNGFRAU-detector setting for ZnO nano-particles. The two insets show, in red, the reconstructed data regions. Plotted data correspond to a single 30 fs shot across all detectors. (*b*) Reconstruction was performed for 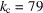, identified as locally optimal by all three performance metrics. (*c*) *S*(*Q*) was calculated for both the reconstructed (red) and gapped (black) patterns, setting gap regions to zero in the latter. (*d*) The corresponding *F*(*Q*) functions show significant effects in the gapped case, due to the zeroed regions in *S*(*Q*). Resultant *G*(*r*) functions show (*e*) extreme artifacts in the gapped case, especially for the low-*r* region, while (*f*) every major feature of the theoretical PDF (red) is present in the PDF corresponding to the reconstructed experimental data (blue). Remaining low-*r* artifacts might be attributed to multi-detector normalization issues and data quality.

A preliminary multi-JUNGFRAU setup, with gaps in the 2θ coverage, was tested but abandoned due to interpolation-related artifacts severely affecting the PDF, most notably for samples with significant diffuse scattering contributions. Fig. 11 ▸(*a*) shows a reconstructed diffraction pattern, collected in such a setting, for ZnO nano-particles. The collected pattern had two gaps in the range 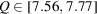 and [13.63, 15.32] Å^−1^, as shown in the insets, with the reconstruction being done for 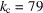. All three of the proposed metrics pointed to this value as locally optimal, as shown in Fig. 11 ▸(*b*). Furthermore, the reconstructed and gapped patterns were both used to calculate experimental PDFs, for comparison. Fig. 11 ▸(*c*) shows the resultant *S*(*Q*) for both cases, with the red curve corresponding to the reconstructed pattern and the black curve to the gapped one. Note how gapped regions in the latter are set to zero, which in turn introduces significant artifacts in the *F*(*Q*) calculation, as shown in Fig. 11 ▸(*d*). This is, of course, a very extreme case, but it highlights the effects of data gaps in PDF analysis. A more reasonable, yet naive, approach would be to perhaps place the zeroed regions in *F*(*Q*), corresponding to filling the gaps in *S*(*Q*) with a constant value of one. Finally, Figs. 11 ▸(*e*) and 11 ▸(*f*) show the resultant *G*(*r*) for both cases, compared with a theoretical PDF calculated for a ZnO nano-particle of similar size. The gapped pattern results in a PDF with extreme effects, especially in the low-*r* region [Fig. 11 ▸(*e*), blue line], while every major feature of the theoretical PDF appears in the PDF corresponding to the reconstructed experimental pattern [Fig. 11 ▸(*f*), blue line]. Beyond some intensity discrepancies and low-*r* effects, which we attribute to multi-detector normalization issues and data quality, the reconstructed pattern allows for the calculation of a reasonable PDF. More importantly, the data shown in Fig. 11 ▸(*a*) correspond to a single 30 fs shot across the JUNGFRAU detectors, which means that a reasonable PDF function has been obtained from a single pulse, despite the presence of gaps in the *Q* space coverage.

Fig. 11 ▸ is thus an example of how the proposed algorithm can naturally be integrated into total scattering and PDF workflows, helping with challenging experimental setups. Furthermore, it highlights the robustness of the proposed metrics to guide the selection of locally optimal cut-off frequency values, and the potential of the method to drastically reduce data collection times. Admittedly, testing in more challenging experimental geometries with larger gaps, more critical gap positions or higher levels of noise is needed.

## 5. Conclusion

A reconstruction procedure for patterns with incomplete information has been presented. It is based on the Papoulis–Gerchberg algorithm but this has been modified to accommodate the particular features of diffraction patterns. The idea behind the iterative algorithm is a filtering in the Fourier transform space of the signal, where the known signal is imposed over the inverse Fourier transform of the filtered pattern. The modifications guide the reconstruction procedure, which stabilizes the solution to physically meaningful minima. The algorithm proved robust to gap position, number of gaps and noise levels, though highly sensitive to the cut-off frequency value. The proposed performance metrics proved useful when dealing with this sensitivity, guiding the selection of locally optimal cut-off frequency values. Tests performed on simulated and experimental data showed good results in the studied cases. The reconstruction algorithm, and the proposed performance metrics, were shown to be useful in challenging experimental geometries with angular gaps.

## Data Availability

Supporting software and data are available upon request.
